# Volume of tidal gas movement in the nonventilated lung during one-lung ventilation and its relevant factors

**DOI:** 10.1186/s12871-020-0937-x

**Published:** 2020-01-22

**Authors:** Jionglin Wei, Lei Gao, Fafa Sun, Mengting Zhang, Weidong Gu

**Affiliations:** 1 Department of Anaesthesiology, Huadong Hospital, Fudan University, 221 West Yan An road, Jing An District, Shanghai, 200040 China; 2 Shanghai Key Laboratory of Clinical Geriatric Medicine, Shanghai, China

**Keywords:** Tidal gas movement, TGM index, One-lung ventilation, Lung collapse, Double-lumen endobronchial tube, Dynamic lung compliance, Thoracoscopy

## Abstract

**Background:**

The passive ventilation of nonventilated lung results in tidal gas movement (TGM) and thus affects lung collapse. The present study aimed to measure the volume of TGM and to analyse the relevant factors of the TGM index (TGM/body surface area).

**Methods:**

One hundred eight patients scheduled for elective thoracoscopic surgeries were enrolled. Lung isolation was achieved with a double-lumen endobronchial tube (DLT). The paediatric spirometry sensor was connected to the double-lumen connector of the nonventilated lung to measure the volume of TGM during one-lung ventilation (OLV) in the lateral position. The TGM index was calculated. The multiple linear regression was analysed using the TGM index as the dependent variables. Independent variables were also recorded: 1) age, sex, body mass index (BMI); 2) forced vital capacity (FVC), FEV_1_/FVC, minute ventilation volume (MVV); 3) dynamic lung compliance (Cdyn) and peak inspiratory pressure (PIP) during dual lung ventilation; 4) the side of OLV; and 5) whether lung puncture for localization of the pulmonary nodule was performed on the day of surgery. The oxygen concentration in the nonventilated lung was measured at 5 min after OLV, and its correlation with the TGM index was analysed.

**Results:**

The volume of TGM in the nonventilated lung during OLV was 78 [37] mL. The TGM index was 45 [20] mL/m^2^ and was negatively correlated with the oxygen concentration in the nonventilated lung at 5 min after OLV. The multiple linear regression model for the TGM index was deduced as follows: TGM index (mL/m^2^) = C + 12.770 × a − 3.987 × b-1.237 × c-2.664 × d, where C is a constant 95.621 mL/m^2^, a is 1 for males and 0 for females, b is 1 for right OLV and 0 for left OLV, c is BMI (kg/m^2^), and d is PIP (cmH_2_O).

**Conclusions:**

The TGM index is negatively correlated with the oxygen concentration of the nonventilated lung at 5 min after OLV. Sex, side of OLV, BMI and PIP are independently correlated with the TGM index.

**Trial registration:**

This study was registered at ChiCTR (www.chictr.org.cn, ChiCTR1900024220) on July 1, 2019.

## Background

Effective collapse of the nonventilated lung is important for the exposure of critical structures during thoracoscopic surgery [1]. Prior to the thoracic cavity being opened to the atmosphere, positive pressure ventilation of the dependent lung results in mediastinal displacement during one-lung ventilation (OLV) [2]. Subsequently, the transient pressure change in the contralateral hemithorax will cause tidal gas movement (TGM), which is also known as passive ventilation [3–5]. Nitrogen can freely leave from and return to the nonventilated lung during passive ventilation. The presence of nitrogen in the lung serves to delay lung collapse as a consequence of a slower uptake of nitrogen from the lung [4, 6, 7]. By lowering the alveolar partial pressure of oxygen, nitrogen in the lung also results in hypoxic pulmonary vasoconstriction, which may further delay lung collapse [3]. The different volumes of TGM may result in different concentrations of oxygen in the nonventilated lung. As a result, TGM affects gas uptake and lung collapse. However, the volume of TGM in the nonventilated lung and its relevant factors remain unknown.

The present study aimed to measure the volume of the tidal gas passively ventilated into the nonventilated lung and to identify the factors associated with the volume of TGM. Furthermore, a mathematical model was established to predict the volume of TGM using multiple linear regression analysis. In addition, the correlation between the volume of TGM and the oxygen concentration in the nonventilated lung at 5 min after OLV was analysed.

## Methods

After approval of the Ethics Committee of Huadong Hospital affiliated to Fudan University and after obtaining written informed consent, adult patients who were scheduled for elective thoracoscopic surgeries were recruited in our prospective observational study from July 2019 to September 2019. All patients received a lung function test and chest computed tomography (CT) examination preoperatively. Patients with the following conditions were excluded: 1) difficulty in airway management detected by the preoperative assessment, 2) unable to receive double-lumen tube intubation, 3) unable to maintain SpO_2_ < 90% during OLV, 4) lung separation failure, and 5) lung bullae or pleural adhesion confirmed by the preoperative CT.

Electrocardiography (ECG), non-invasive blood pressure (NIBP) and pulse oximetry were monitored in all patients before anaesthesia. One hundred percent oxygen was inhaled during the induction of anaesthesia and measurement of the volume of TGM.

All patients received total intravenous anaesthesia [8]. Anaesthesia was induced with propofol (1–2 mg/kg), sufentanil (0.3 μg/kg), and rocuronium (1 mg/kg) and was maintained with an infusion of propofol (100–150 μg/kg·min) and remifentanil (0.15–0.2 μg/kg·min). Intermittent boluses of rocuronium were injected according to a train-of-four neuromuscular monitoring.

The tidal volume was set as 7 mL/kg (ideal body weight) during two-lung ventilation and 6 mL/kg (ideal body weight) during OLV, respectively. The respiratory rate was 12/min, and the I:E ratio was 1:2 without positive end-expiratory pressure (PEEP) [9, 10].

Lung isolation was achieved with an appropriately sized left-sided double-lumen endobronchial tube (DLT) [11]. Before placement of the DLT, fibreoptic bronchoscopy examination was performed to inspect the bronchial anatomy and pathology and to remove secretions. The position of the DLT was confirmed under fibre bronchoscopy. Dynamic lung compliance (Cdyn) and peak inspiratory pressure (PIP) during dual-lung ventilation were measured three times, and the average values were calculated for the analysis. The patients were turned to the lateral position when the exhaled oxygen concentration reached 90%. The position of DLT was reconfirmed and adjusted as needed under fibreoptic bronchoscopy [12, 13]. OLV of the dependent lung was started immediately after the placement of DLT. The fraction of the oxygen concentration in the nonventilated lung was measured at 5 min after OLV using a gas concentration monitor (S/5 Anaesthesia Monitor, GE, Finland). In brief, the nonventilated lung was mechanically ventilated at 5 min after OLV. End-expiratory oxygen concentration during the first mechanical ventilation was recorded as the fraction of the oxygen concentration in the nonventilated lung at 5 min after OLV. The accurate location of the DLT was important for lung isolation. If the difference between the actual tidal volumes and the pre-set tidal volumes was more than 10 mL, the patient had to be excluded due to lung separation failure. All procedures were performed by one anaesthesiologist (J.W.).

The volume of TGM in the nonventilated lung was measured in the lateral position using a paediatric spirometry sensor (Pedi-lite Sensor, REF73393, GE, Finland). The sensor was connected to the double-lumen connector of the nonventilated lung [14] which was open to the atmosphere. When the OLV was started, the volume of TGM was consecutively measured three times. The average value was calculated. To increase the comparability between different races, the TGM index (ratio of volume of TGM to body surface area [15]) was calculated and analysed.

The following variables were also recorded: 1) age, sex, body mass index (BMI); 2) forced vital capacity (FVC), FEV1/FVC, minute ventilation volume (MVV) from the pulmonary function test; 3) Cdyn and PIP during dual lung ventilation; 4) side of OLV (left/right); and 5) lung puncture for the localization of the pulmonary nodule on the day of surgery (yes/no).

### Statistical analysis

Data were analysed using the SPSS statistical software package (version 23.0; IBM, Chicago, IL, USA). The TGM index was defined as a continuous outcome variable (dependent variable). Other variables (independent variables) were shown according to the following rules. Continuous variables were analysed with the Shapiro-Wilk test for normality. Normally distributed variables were expressed as the mean (standard deviation), and nonnormally distributed variables were expressed as the median [interquartile range]. Binomial variables were expressed as numbers (percentages).

All data were analysed by the single factor correlation analysis. For normally distributed continuous variables, a correlation analysis was performed with Pearson correlation test, while nonnormally distributed data were analysed with Spearman correlation test. Binomial variables were analysed with Student’s t-test.

Variables with *P* < 0.25 were selected for the subsequent multiple linear regression analysis. Statistical differences were considered to be significant if *P* < 0.05.

### Sample size estimation

Sample size was estimated according to the rule-of-thumb of *N* ≥ 50 + 8 *m*, where *m* is the number of independent variables [16]. In our pilot study, multiple linear regression analysis showed that there were 4 independent variables. Therefore, the minimum sample size was 82 in the present study.

## Results

**Fig. 1:**
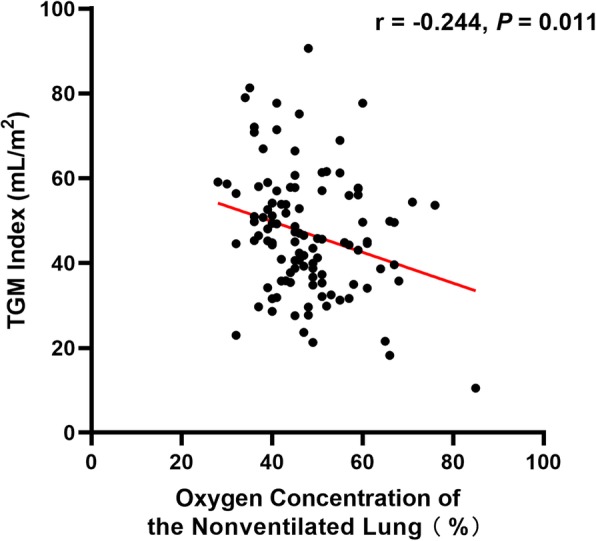
Negative correlation between the TGM index and oxygen concentration in the nonventilated lung at 5 min after OLV. TGM index, ratio of volume of tidal gas movement to body surface area. OLV, one-lung ventilation

One hundred eight patients were enrolled. The volume of TGM was 78 [37] mL. The TGM index was 45 [20] mL/m^2^. There was a negative correlation between the TGM index and oxygen concentration in the nonventilated lung at 5 min after OLV (*r* = − 0.244, *P* = 0.011) (Fig. 1).

**Fig. 2:**
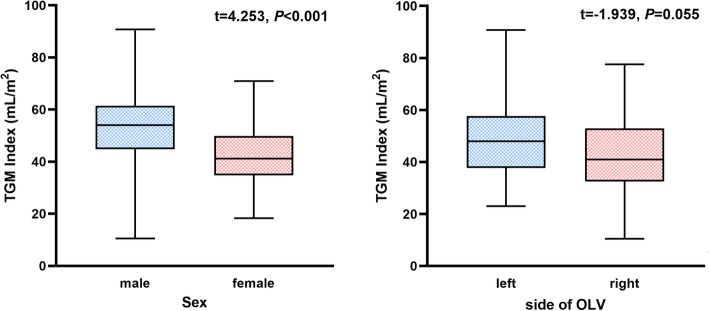
The correlation between the TGM index and sex or side of OLV, which were two of the binary classification variables, based on multiple linear regression analysis and box plots. The box shows the interquartile range (IQR) and the median of each variable. TGM index, ratio of volume of tidal gas movement to body surface area. OLV, one-lung ventilation

**Fig. 3:**
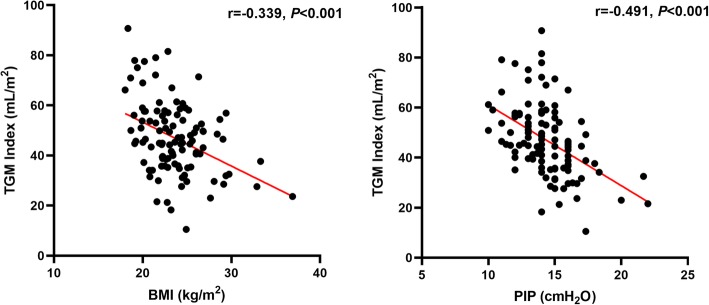
The correlation between the TGM index and BMI or PIP based on multiple linear regression analysis. TGM index, ratio of volume of tidal gas movement to body surface area. BMI, body mass index. PIP, peak inspiratory pressure

There was a significant correlation between the TGM index and sex, BMI, FVC, Cdyn or PIP (Table 1, *P* < 0.05) (Fig. 2, Fig. 3).

**Table 1:** Results of single-factor correlation analysis

	Distribution	*P* value
Age (y), mean (SD)	56 (12)	0.051
Sex (female/male), N	63/45	< 0.001
BMI (kg/m^2^), median [IQR]	24 [4]	< 0.001
Pulmonary function tests		
FVC (L), median [IQR]	2.6 [0.9]	< 0.001
MVV (L/min), median [IQR]	29 [19]	0.610
FEV_1_/FVC (%), median [IQR]	89 [10]	0.752
Pulmonary dynamics during two-lung ventilation		
Cdyn (mL/cmH_2_O), mean (SD)	48 (13)	< 0.001
PIP (cmH_2_O), median [IQR]	14.0 [2.7]	< 0.001
Side of one-lung ventilation (right/left), N	39/69	0.055
Lung puncture on the surgery day (yes/no), N	40/68	0.659

Continuous data are shown as the mean (SD) or median [IQR] according to normality tests. Binary classification data are shown as N

*SD* standard deviation; *IQR* interquartile range; *N* number

*BMI* body mass index; *BMI* weight in kilograms divided by height in metres squared; *FVC* forced vital capacity; *MVV* minute ventilation volume; *FEV1* forced expiratory volume at 1 s; *Cdyn* dynamic lung compliance; *PIP* peak inspiratory pressure

Seven variables with *P* < 0.25, including sex, age, BMI, FVC, Cdyn, PIP and side of OLV, were selected to perform the multiple linear regression analysis. Sex, side of OLV, BMI and PIP were independently correlated with the TGM index (Table 2, *P* < 0.05). The unstandardized coefficients were shown in Table 2. B values were the partial regression coefficients. The constant of the regression model was 95.621 mL/m^2^. According to the multiple linear regression analysis, a linear regression model was deduced for the TGM index as follows:

**Table 2:** Results of the multiple linear regression analysis

	Unstandardized Coefficients		Standardized Coefficients			95% CI for B	
	B	Std. Error	Beta	t	*P* value	Lower	Upper
Constant	95.621	17.437		5.484	< 0.001	61.026	130.216
Sex (male)	12.770	3.134	0.434	4.075	< 0.001	6.552	18.987
OLV (right)	-3.987	1.961	−0.132	−2.033	0.045	−7.878	− 0.097
BMI (kg/m^2^)	−1.237	0.338	−0.277	−3.660	< 0.001	− 1.907	− 0.566
PIP (mean) (cmH_2_O)	−2.664	0.710	−0.388	−3.754	< 0.001	−4.072	−1.256

*OLV* one-lung ventilation; *BMI* body mass index; *PIP* peak inspiratory pressure

TGM index (mL/m^2^) = C + 12.770 × a-3.987 × b-1.237 × c-2.664 × d.

C: Constant = 95.621;

a: male = 1, female = 0;

b: right OLV = 1, left OLV = 0;

c: BMI (kg/m^2^);

d: PIP (cmH_2_O).

## Discussion

The present study confirmed that transient changes in the pleural pressure in the nonventilated lung exist during one-lung positive pressure ventilation before opening the thoracic cavity of the nonventilated lung. As a result, it caused tidal movement of gas out of and back into the nonventilated lung. The volume of TGM in the nonventilated lung in the lateral position ranged from 19 to 160 mL.

The measurement of the volume of TGM was performed using a paediatric spirometry sensor. The measurement range was 5 to 300 mL with an accuracy of ±6% or 4 mL. It is more accurate to measure small gas movements with a paediatric spirometry senor than with an adult spirometer. Previous studies used different tools to measure the volume of TGM, such as a potentiometer attached to the counter-balance wheel of the spirometer [2], a water-filled spirometer [3], or an ambient pressure oxygen reservoir bag apparatus [4]. Compared to the above methods, the paediatric spirometry sensor used in the present study makes the measurement simpler and more accurate. In addition, the volume of TGM can be measured in real-time with a repeatable way by using the paediatric spirometry sensor. The volume of TGM in the present study was smaller than those in other studies (65–265 mL) [3]. This result may be related to race differences. Therefore, we calculated the TGM index, which allows for comparing TGM between individuals of different sizes.

The oxygen concentration in the nonventilated lung at 5 min after OLV was negatively correlated with the TGM index in the present study. This could be the result of the nitrogen going into the nonventilated lung by passive ventilation and diluting the oxygen concentration. Since nitrogen is absorbed slower than oxygen, the results suggested that a higher TGM volume may delay lung collapse.

Since there is no research on the analysis of factors associated with the TGM index, ten variables were chosen for the single factor correlation analysis. These variables are the most commonly used clinical indicators and include the patients’ general clinical information, lung function variables and basic anaesthesia-related factors. According to the multiple linear regression analysis, sex, side of OLV, BMI and PIP were independently correlated with the TGM index. To the best of our knowledge, this is the first time that a regression model was established to predict the TGM index. Using this model, anaesthesiologists can calculate the TGM index before surgery and evaluate the impact of TGM on the collapse of the nonventilated lung.

In the present study, male patients had a larger TGM index than female patients. Sex was independently correlated with the TGM index. The B value of sex was 12.770 mL/m^2^. The underlying reason may be related to anatomical differences. The lung volume of adult females is typically 10–12% smaller than that of males who have the same height and age as females. It results from the differences in the thoracic dimensions between males and females. Because of a greater inclination of ribs, female rib cages could accommodate for a greater volume expansion [17]. During OLV, there is more space for the ventilated lung to expand, less mediastinal movement and less TGM of the nonventilated lung in female patients.

The side of OLV was also independently correlated with the TGM index. Patients with right side OLV produced a lower volume of TGM than those with left side OLV. This may be caused by the anatomical differences between the left and right lungs. The volume of the right lung is larger than that of the left lung, which shares space in the chest with the heart. The right lung has three lobes with 10 segments, while the left lung has only two lobes with 8 segments. Since the tidal volume was the same between left OLV and right OLV, the mediastinal movement was more significant during left OLV than during right OLV. Therefore, the volume of TGM was larger during left OLV than during right OLV.

In the present study, BMI was negatively correlated with the TGM index. The B value is − 1.237. BMI is an important determinant of respiratory function, especially in obese patients [18]. The patients with a higher BMI have low pulmonary/chest wall compliance and increased airway resistance. In addition, there is more adipose tissue in the mediastinum in obese patients. These will consequently make the mediastinal movement more difficult.

PIP depends on airway resistance and pulmonary/chest wall compliance. In the present study, PIP was measured during dual lung ventilation immediately after DLT intubation. The results indicated that the PIP was negatively correlated with the TGM index. The B value was − 2.664. The lung with less PIP was associated with more TGM in the nonventilated lung under the same external force.

There were several limitations in the present study. First, as a single-centre observational study, the sample size was relatively small. Multicentre, large sample observation studies should be performed in the future. Second, TGM was measured only when the tidal volume was set as 6 mL/kg of ideal body weight during OLV. Although 6 mL/kg of ideal body weight is the commonly used tide volume during OLV, the presented regression model may be unsuitable for other tidal volumes. Third, TGM may indirectly affect oxygenation during surgery by delaying lung collapse. Therefore, the relationship between TGM and oxygenation during surgery needs to be further investigated.

In conclusion, TGM occurs in the nonventilated lung during one-lung ventilation. The TGM index is negatively correlated with the oxygen concentration in the nonventilated lung at 5 min after OLV. A greater volume of TGM might delay lung collapse during thoracic surgery. Sex, FVC, BMI and PIP are independently correlated with the TGM index. The regression model in the present study may be used to predict the TGM index before surgery and to guide OLV management.

## Data Availability

The raw data of the current study are available from the corresponding author on reasonable request.

## Abbreviations

BMI: Body mass index
BSA: Body surface area
Cdyn: Dynamic lung compliance
DLT: Double-lumen tube
FEV1: Forced expiratory volume in the first second
FVC: Forced vital capacity
MVV: Minute ventilation volume
OLV: One-lung ventilation
PEEP: Positive end-expiratory pressure
PIP: Peak inspiratory pressure
TGM: Tidal gas movement
